# Telemedicine for new immigrant children in the US: a tool for equity or another layer of disparity?

**DOI:** 10.3389/fped.2026.1814069

**Published:** 2026-04-30

**Authors:** Niriksha Ravi, Michael Olaseni Bamgbose, Yousra Yehia Abdelkhalek, Saloni Parkar, Yossef Alnasser

**Affiliations:** 1 Department of Pediatrics, BronxCare Health System, Icahn School of Medicine at Mount Sinai, New York, NY, United States; 2 Department of Biological Sciences, College of Science, Purdue University, West Lafayette, IN, United States

**Keywords:** digital divide, health equity, healthcare access, new immigrants, telemedicine

## Abstract

Telemedicine refers to the use of digital communication tools to deliver healthcare services. In pediatrics care in the United States (US), it has become an important approach to expand access to primary and subspecialty care while reducing travel demands and improving continuity of care, particularly for children in remote and underserved communities. Evidence consistently shows that telemedicine can supplement in-person visits while maintaining high levels of family satisfaction and clinical effectiveness among American families. This narrative review was developed to assess the applicability of telemedicine for new immigrant families in the US using a structured literature search across PubMed, Scopus, Google Scholar, and Cochrane of published English literature. Peer-reviewed studies were included if they were conducted in the last 50 years and focused on pediatric patients from birth to 21 years of age. Findings were synthesized to evaluate the acceptance, accessibility, feasibility, and applicability of telemedicine for new immigrant children in the US. Across the reviewed literature, telemedicine has meaningful potential in narrowing health disparities faced by new immigrant families such as transportation, specialist shortages, scheduling delays, limited English proficiency, and difficulty navigating the complex healthcare system. Furthermore, telemedicine can connect families with providers who share similar cultural and linguistic backgrounds, thereby strengthening cultural sensitivity and trust. Still, it comes with several obstacles limiting its use among new immigrant families. From digital divide, poor network coverage, low health literacy, privacy concerns, immigration-related concerns, to mistrust, the challenges are numerous. Studies during the COVID-19 pandemic found low acceptance and infeasibility of telemedicine for new immigrant families. However, telemedicine offers multiple opportunities and future directions to better serve immigrant children and their families. Expanding multilingual telehealth platforms and integrating telemedicine access points into schools, community centers, and immigrant resource hubs can enhance accessibility, usability, and acceptance. Pairing telemedicine with artificial intelligence can have huge future potential and might be a tool for inclusive care at lower cost. Furthermore, policy amendments, particularly broader Medicaid telemedicine coverage and mandates for interpreter integration into telemedicine workflows, are essential for promoting more equitable access with higher acceptance and more applicability for new immigrant children in the US.

## Introduction

Telemedicine refers to the use of digital communication technologies, including video visits, telephone consultations, and mobile health to deliver healthcare services remotely (1–3). In pediatric care, telemedicine has become an increasingly common component of clinical practice, supporting routine visits, specialty consultations, and follow-up care (1–4). Its adoption accelerated rapidly in the US during the COVID-19 pandemic, as healthcare systems sought to preserve access to pediatric services while minimizing in-person encounters. The pandemic taught us that telemedicine can serve as both a supplement and alternative to in-person pediatric visits (3, 5–7). Telemedicine refers specifically to remote clinical care, whereas telehealth is a broader term that includes telemedicine along with non-clinical services such as education and administrative support (7).

Evidence suggests that telemedicine can maintain clinical effectiveness while improving convenience and continuity of care for American children and their families (1, 4, 5). Pediatric telemedicine has been associated with reduced travel burden, improved access to specialty services, shorter wait times, and timely clinical decision-making without compromising patient safety (1, 5, 8, 9). These advantages have contributed to widespread adoption across pediatric healthcare settings (2, 6). Still, the applicability and acceptance by new immigrant children and families are still under-investigated. Furthermore,

new immigrant children are children whom recently arrived in the United States within the last 5 years (10). Immigrant populations frequently encounter structural barriers such as lack of insurance, transportation limitations, language discordance, and challenges navigating the US healthcare system (10–14).

Telemedicine has the potential to mitigate some of these barriers, although some disparities in telemedicine utilization might exist (15–19). Still, emerging evidence supports the clinical effectiveness of telemedicine in pediatric care. A recent systematic review and meta-analysis found that telemedicine use in pediatric emergencies was associated with reduced hospital length of stay, lower overall mortality, and comparable admission rates to in-person care (20, 21). Overall, these findings highlight both the promise and limitations of telemedicine for new immigrant children and underscore the importance of evaluating its applicability and feasibility for new immigrant children and their families in the US (21, 22). Therefore, this narrative review is set to summarize the current literature about utility, accessibility, acceptance, feasibility and applicability of pediatric telemedicine for new immigrant children in the US.

## Methods

### Search engine and data selection

This narrative review was conducted using a structured literature search across PubMed, Scopus, Google Scholar, and Cochrane databases over the last 50 years. Search terms included singular and combinations of keywords, including telemedicine, telehealth, pediatrics, new immigrant children, healthcare access, health disparities, digital divide, health policy, insurance, digital divid and digital equity. Boolean operators (AND/OR) were used to refine the search strategy. Given this is a narrative review, authors aimed to include significant and recent data, but cannot claim to summarize the whole literature due to the lack of a systematic review nature of this work.

### Criteria

Titles and abstracts were screened for relevance, followed by a full-text review of selected articles to be included in this narrative review.

Studies were included if they were peer-reviewed, written in English, conducted in the US over the last 50years, and focused on pediatric patients from birth to 21 years of age. The 50-year timeline was chosen in accordance with the establishment of telemedicine in the US (19). Articles specifically examining new immigrant populations, language barriers, digital access, interpreter services, or health equity in telemedicine were prioritized to be included in this narrative review. Studies were excluded if they focused exclusively on adult populations, were conducted outside the United States, were not peer-reviewed, or did not address healthcare delivery through telemedicine. Reference lists of included articles were reviewed to identify additional relevant studies. Still, this review might have missed some literature due to a lack of systematic literature screening of this review.

### Data synthesis

A broad range of studies were included based on relevance to the new immigrant children. Findings were synthesized qualitatively through thematic analysis of five main themes following established narrative review methodology framework (22). The six main themes were chosen after consulting with two experts in pediatric telemedicine in the US and included: Accessibility, acceptance, feasibility, applicability, barriers, and Opportunities.

## Results and discussion

### Can telemedicine improve access for new immigrant children to healthcare in the US?

Telemedicine has demonstrated strong potential to increase healthcare access for new immigrant children and their families (2, 5, 8, 19). A school-based mental health intervention delivered via telemedicine for newcomer immigrant children reported improved access to care for both participants and providers while reducing stigma (23–26). This improvement was attributed to reduced transportation needs, fewer space constraints, and increased flexibility enabled by digital platforms (23–28). Participants also described enhanced engagement and comfort in sharing emotions with enhanced privacy options through features such as chat functions or camera-off options, supporting participation among emotionally vulnerable youth (23).

Telemedicine with expanded scheduling flexibility to include evening and weekend appointments was found to be a significant facilitator of access to care for new immigrant families with working caregivers who face challenges attending traditional in-person visits (2, 24). Similarly, telemedicine improved access to mass developmental screening through telephone-based and virtual models, with higher screening completion rates observed among families facing logistical barriers (29, 30). Cost savings represent another important advantage of telemedicine while improving access to care, particularly when accounting for direct and indirect costs (5, 9, 31). Telemedicine visits are often shorter and more flexible while still allowing clinicians to identify medical concerns and initiate appropriate follow-up care (8, 16). This can allow providers to see more patients and have a higher impact on such a vulnerable population (8, 16). When thoughtfully implemented, telemedicine has demonstrated the capacity to improve equitable access across pediatric populations regardless of age, diagnosis, or language background (6, 16, 19, 26). However, telemedicine is not able to overcome some structural barriers to access pediatric care in the US, like a lack of insurance, a fragmented healthcare system, and distrust. Still, it can provide alternative routes to access care during times of uncertainty, like what happened during the COVID-19 pandemic (6, 11, 16).

### Do new immigrant families accept telemedicine?

Despite its benefits, telemedicine acceptance among new immigrant families is shaped by multiple factors. These include digital divide factors stemming from unreliable internet access, limited availability of digital devices, limited digital literacy and inconsistent teleservices (23, 32–35). Studies indicate that a meaningful proportion of immigrant families lack consistent access to smartphones, broadband, or data plans due to financial constraints, limiting their ability to participate in telemedicine visits (20, 24, 36, 37). Additionally, other factors include difficulty establishing rapport with clinicians, lack of representation, and lack of private spaces within the place of residence for confidential virtual visits (23, 32–35).

Health literacy and trust also influence acceptance. Some families remain unaware that telemedicine is a legally and medically recognized form of healthcare (6, 11, 16). Other families thought telehealth services cannot be accessed via smartphones and need computers, which they do not own (29, 38). Concerns about perceived quality of care and diagnostic limitations further influence acceptance, particularly in communities with historical experiences of being marginalized (4, 30, 39). On the other hand, telemedicine utilization has been shown to be higher among English-speaking families and those with commercial insurance, while families with limited English proficiency, public insurance, or interpreter needs are less likely to accept, initiate or complete telemedicine visits (17–19, 35, 40, 41).

Provider's comfort and workflow adaptation can shape acceptance, as clinicians vary in their experience with virtual care and perceptions of its diagnostic adequacy (4, 32). Although home-based testing and remote monitoring technologies continue to expand, limitations in physical examination remain a concern for both families and providers to accept telemedicine (4, 42). Interventions that provide new immigrant families with digital devices, broadband access, and technical support have been shown to improve telemedicine uptake, acceptance, and accessibility (27, 29, 34, 38, 43). Still, acceptance of pediatric telemedicine among many new immigrant families is a major barrier to scaling up this service to be a tool for health equity.

### How feasible is telemedicine for new immigrant children and families in the US?

The feasibility of telemedicine for new immigrant children and their families depends on technological access, language support, digital literacy, and healthcare system design. While telemedicine offers logistical advantages such as reduced travel time and increased appointment flexibility, these benefits are not equitably realized. Evidence suggests that telemedicine may inadvertently exacerbate disparities for families with limited English proficiency when interpreter services and language-concordant platforms are not fully integrated into virtual care delivery (17–19, 44).

Language discordance remains a central barrier. Patients with limited English proficiency experience poorer health outcomes and lower satisfaction in traditional clinical settings, challenges that may be magnified in virtual environments (17, 45–47). Telemedicine platforms and patient portals are frequently available only in English, creating additional obstacles for caregivers attempting to schedule appointments, complete consent forms, or follow visit instructions (11, 43). Missed appointments and communication breakdowns occur more frequently among non-English-speaking families during virtual visits (17, 48).

Digital literacy and structural inequities further influence feasibility. Mixed-methods studies examining telehealth use among new immigrant populations demonstrate that limited English proficiency and low digital health literacy are strongly associated with reduced telemedicine utilization and delayed care (11, 19, 44, 49). Participants describe challenges navigating telehealth platforms, concerns about privacy and data security, and unfamiliarity with virtual healthcare workflows (33, 34, 49). Healthcare system analyses, including studies of pediatric Medicaid populations, reveal persistent disparities in telemedicine utilization by race, ethnicity, and socioeconomic status despite expanded provider availability (20, 35, 50). Despite all the advantages of telemedicine in overcoming many barriers to ease access to care, it cannot be offered without insurance coverage. Even with insurance coverage, low intake of telemedicine has been reported in the literature among new immigrant families due to digital divide (43). Therefore, not all forms of pediatric telemedicine are feasible for all new immigrant families in the US.

### Barriers and challenges of using telemedicine for new immigrant children

Children from immigrant families are disproportionately affected by socioeconomic disadvantages that limit access to healthcare, including telemedicine services (10–14). Studies demonstrate that multiple interrelated factors, including language barriers, limited digital access, and inadequate interpreter availability, play a critical role in shaping telemedicine utilization, with the potential to worsen existing inequalities if left unaddressed (15–20).

Key barriers faced by new immigrants include limited access to digital devices, unreliable internet connectivity, limited English proficiency, privacy concerns, and fear related to immigration enforcement or misuse of personal data (10–12, 33, 34, 39). Effective telemedicine implementation depends on a reliable technological infrastructure, stable internet connectivity, and familiarity with digital tools, which are often lacking in low-income immigrant households (11, 19, 36, 44). A Latinx immigrant population in a study by Hodges JC et al. utilize telemedicine at lower rates. These populations are less likely to own desktop or tablet computers or smartphones and are less likely to have home broadband internet (40). Many newly arrived immigrant families are under-connected, relying on shared devices, slow or unstable connections, financial trade-offs, or mobile-only data plans (3). These limitations hinder consistent engagement with telemedicine and further reduce immigrant children's ability to benefit from telemedicine services.

Language barriers further restrict telemedicine use, particularly for families who speak languages other than English or Spanish. Studies demonstrate lower telemedicine utilization and poorer experiences among families requiring interpreter services, especially when interpreter integration is inconsistent or delayed (18, 44, 47, 48). A study by Pathak et al. involving four community-based pediatric practices in New York found that non-English speaking families had significantly lower odds of patient portal activation and video visit utilization, with the lowest rates observed among families who spoke neither English nor Spanish (34).

**Figure 1:**
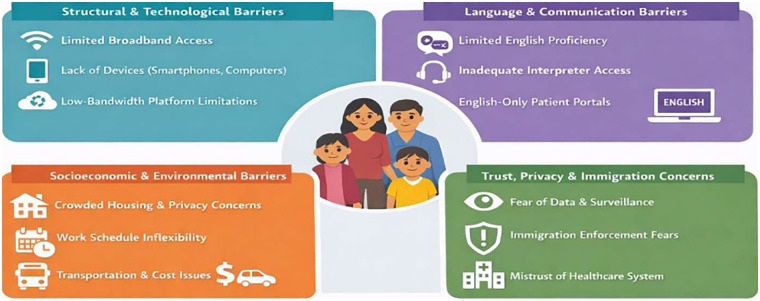
Multilevel barriers to telemedicine use among new immigrant children and their families.

Privacy concerns are especially salient for families living in crowded or shared housing environments, where confidential conversations may be overheard (33, 34). Fear of immigration enforcement and concerns about digital surveillance may also lead families to delay or avoid telemedicine encounters altogether (12, 39, 42) Figure 1.

Healthcare access for new immigrant children is significantly affected by public insurance policies as many must meet a 5-year residency requirement before qualifying for Medicaid and Children's Health Insurance Program Reauthorization Act (CHIP). Expansion under the CHIP allowed states to waive this waiting period through federal options, thereby enabling earlier enrollment and improving access in some states. However, persistence in state-level variation continues to drive disparities in healthcare. Overall, policy-level barriers, especially insurance eligibility, are the biggest hurdle to offering and utilizing pediatric telemedicine for many new immigrant families in the US.

### Opportunities and future directions for telemedicine for new immigrant children

Telemedicine offers meaningful opportunities to improve healthcare access for new immigrant children. Integrating professional interpreter services directly into telemedicine platforms improves communication quality, patient satisfaction, and clinical outcomes (10, 18, 34, 44, 46, 47). Simplified, multilingual patient portals and culturally tailored onboarding processes further enhance accessibility and engagement (11, 25, 43).

Integration of artificial intelligence (AI) with telemedicine represents a promising future direction for new immigrant children in the US. Telemedicine combined with AI can help in improving access and quality of patient care, particularly for underserved and new immigrant populations (1–6, 11, 19, 24). AI-enabled tools enhance decision-making, improve care coordination and help streamline workflow. In addition, they help address some limitations like communication barriers and workforce shortages (8, 16, 28, 32, 44). However, integration of AI in telemedicine will require careful attention to digital literacy, data privacy, and algorithmic bias to ensure that technological advances do not exacerbate existing health care inequities (6, 11, 24, 33).

Low-bandwidth, mobile-friendly telemedicine platforms and delivery models embedded within trusted settings such as school-based health centers and community clinics can improve utilization among new immigrant families (25–28). Culturally responsive enrollment strategies, involvement of linguistically concordant healthcare staff, and partnerships with community organizations help build trust and reduce perceived barriers (2, 13, 14). For undocumented immigrant children, strong confidentiality protections, transparent data-use policies, and routine privacy check-ins are essential to improving comfort and utilization of telemedicine services (12, 33, 39, 42, 51, 52) Table 1.

**Table 1:** Telemedicine: insight, evidence, gaps and future prospects.

Domain	What current evidence depicts	Key gaps identified	Implications for future research
Can Telemedicine Improve Access for New Immigrant Children to Healthcare in the US?	Reducing travel. Scheduling flexibility. Lowering indirect costs. Increasing access to mental health and developmental services for new immigrant children.	Limited longitudinal outcome data. Not inclusive of all new immigrant children	Need for long-term, outcome-focused studies assessing disease control, preventive care uptake, and continuity of care in new immigrant pediatric populations (2, 5, 6, 8, 9, 11, 16, 19, 23–31).
Do New Immigrant Families Accept Telemedicine?	High acceptance for technological support, language services. Families value convenience and reduced disruption to work and school.	Acceptance varies by language proficiency, digital literacy, and trust in healthcare. Limited data for non-Spanish speakers.	Studies examining trust, cultural perceptions, and caregiver preferences across diverse immigrant communities (4, 6, 11, 16, 20, 23, 24, 32–43).
How Feasible Is Telemedicine for New Immigrant Children and Families in the US?	Improves with interpreter services, mobile-friendly platforms, and institutional support. Feasible in primary and mental healthcare settings.	Utilization disparities among low-income families. Workflow challenges for interpreters and providers.	Research on system-level interventions, multilingual platforms, and reimbursement models that improve feasibility and equity. (10–15, 18–20, 33, 34, 36, 39, 40, 42, 44, 47, 48).
Opportunities and Future Directions for Telemedicine for New Immigrant Children	Community partnerships, school-based telehealth. Low-bandwidth platforms. Culturally responsive care models. AI in telemedicine.	Limited scalability data. Unclear sustainability Few policy evaluations specific to immigrant children.	Implementation studies to identify scalable, sustainable telemedicine models tailored to new immigrant families. (1–6, 10–14, 16, 18, 19, 24–28, 33, 34, 39, 42, 44, 51, 52).

## Conclusion

Telemedicine represents a promising strategy to improve healthcare access for new immigrant children in the US by ability to help in reducing some logistical, financial, and temporal barriers to care. Evidence demonstrates that telemedicine can enhance access to primary, specialized, and preventive services while maintaining clinical effectiveness. However, persistent structural challenges including lack of insurance, limited English proficiency, digital literacy gaps, socioeconomic constraints, and privacy concerns continue to limit equitable utilization among new immigrant families. Furthermore, the lack of acceptance of telemedicine by new immigrant families remains a major barrier.

Expanding Medicaid and commercial insurance coverage for all forms of telemedicine can improve accessibility to pediatric telemedicine services in the US for new immigrant families. To ensure telemedicine fulfills its potential as a tool for health equity, healthcare systems must intentionally integrate interpreter services, culturally responsive care models, enhance health literacy and digital access support into virtual care delivery to enhance acceptance, feasibility, and applicability of telemedicine among new immigrant families in the US. Policy initiatives and institutional investments aimed at improving broadband access, device availability, and digital literacy are also essential.
